# Decoupling what, how, and when for observing decision-making context in autonomous robots

**DOI:** 10.3389/frobt.2026.1847701

**Published:** 2026-07-22

**Authors:** Johannes Ernst, David Lennart Risch, Peter Lehner, Freek Stulp, Andreas Dömel

**Affiliations:** Robotics and Mechatronics Center (RMC), German Aerospace Center (DLR), Weßling, Germany

**Keywords:** autonomous agents, behavior trees, context-dependent execution, mobile manipulation, situation awareness, software architecture for robotic and automation

## Abstract

This paper presents an approach that decouples what to observe, how to observe it, and when observations are required for decision-making in autonomous robots. Situation awareness is essential for efficient and reliable autonomous robot operation, but despite advances toward parallelizing perception and action, key challenges remain in making perception aware of the current context and ensuring observability during action execution. To address this, we explicitly model the decision-making context and identify it with dedicated observers running in parallel to the task execution. Observer behaviors are implemented as behavior trees and coordinated by a centralized context manager. We validate the approach on a mobile manipulator that performs autonomous machine-tending tasks in a real medical laboratory, as well as during a public trade fair. Experimental results show that context-driven observers can robustly identify decision-making context without interfering with ongoing actions. Furthermore, parallelized perception leads to substantial runtime improvements, achieving overall task time savings of up to 24% . These findings demonstrate that explicit context modeling and observer-based perception parallelization enhance the stability and efficiency of existing robotic execution architectures.

## 1 Introduction

**FIGURE 1:**
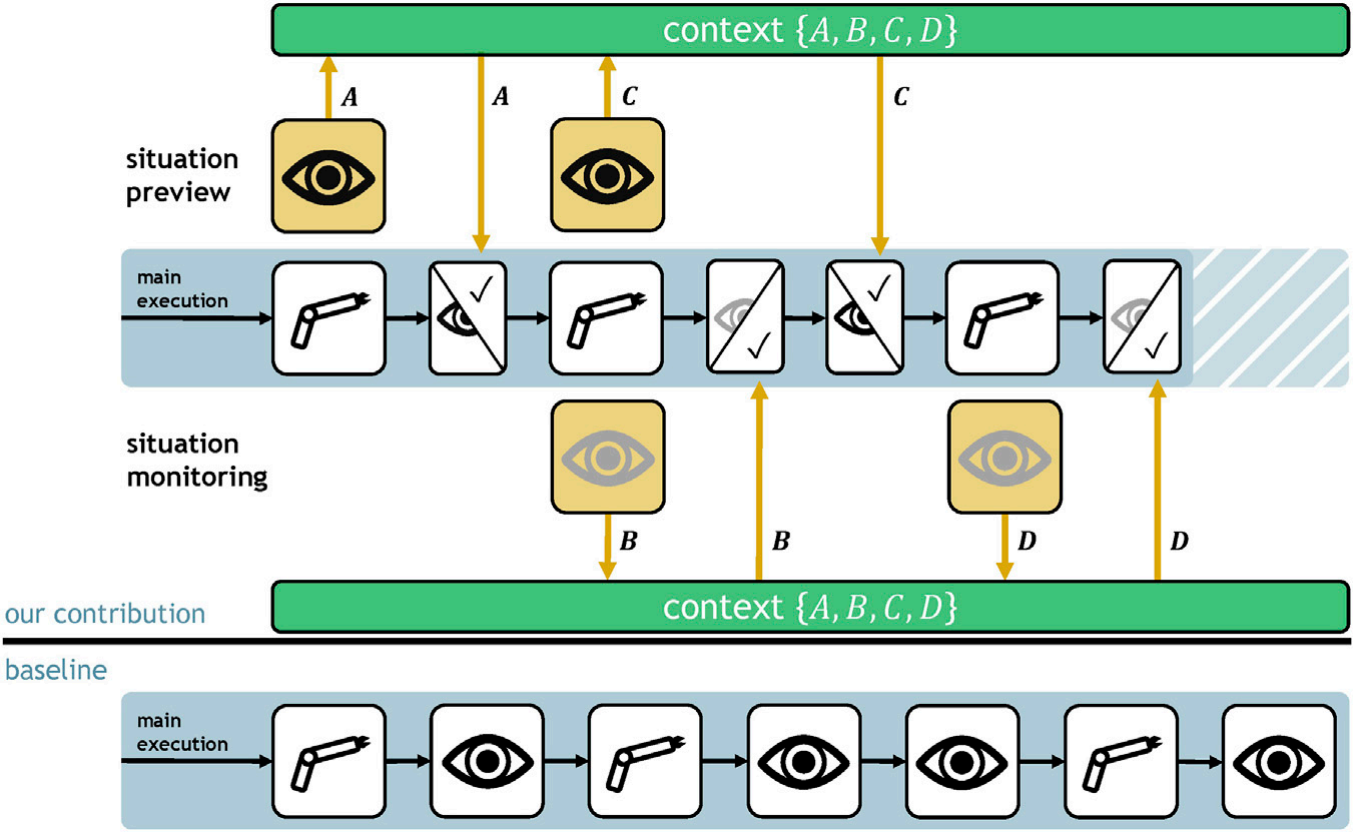
Parallelization of perception and action to increase the situation awareness. Sequential perception is decoupled from the main execution and dragged forward in time for situation preview and monitoring. Situation preview describes relevant context observations in advance of upcoming actions while situation monitoring describes the parallel context observation of ongoing actions. The observed context is then used for decision-making in the main execution.

The sense-plan-act paradigm has been central to the development of autonomous robots since the early days of robotics and still influences their behavior (Kortenkamp et al., 2016). Information is gathered via the system’s perception and then used to plan and execute an action. As shown in the middle of Figure 1, this approach results in a sequence of alternating perception and action blocks. The task of perception is to determine the relevant information for the subsequent action, or to check the successful execution of a previous action. The action, in turn, determines what information is necessary. In addition, actions influence how the situation changes for the next perception.

The sense-plan-act paradigm has two major limitations: First, it is inefficient. If, for example, another object is to be grasped, it is necessary to wait for perception again, even though the object has not moved and the situation is unchanged. Even more problematic is the reduced robustness to changes in the environment. If the object is moved unexpectedly during the action, the robot does not notice.

Both problems have a common cause: A lack of situation awareness (Smith and Hancock, 1995) (for term definitions, see Section 2). If the situation has not changed but perception must still be initiated, it means that the robot is not aware of the situation’s steadiness at the relevant time. If the situation changes, but the robot cannot recognize it because it is in the middle of an action, it also lacks knowledge about the current situation.

Our goal is to increase the situation awareness of sense-plan-act systems by decoupling the what, how, and when of context observation from the main execution. This allows for parallelization of perception and action by providing the relevant contextual information when it is needed. As shown in Figure 1, we distinguish *situation preview* and *situation monitoring*. The former addresses the perception that is needed for the following action. The latter, on the other hand, addresses the perception that monitors the successful execution of a simultaneous action. In both cases, the perception blocks are moved forward in time to run in parallel to the main execution’s actions, leading to a more efficient execution.

The parallelization of perception and action poses several challenges. The first challenge is to determine *what* specific perception results are needed *when* during the main execution for decision-making. Even more challenging is *how* to parallelize perception while other actions take place. For sense-plan-act architectures, perception is used in specific situations where the observability of the context is usually ensured. When perception is parallelized, the observability of a context might vary due to ongoing action. For example, the robot manipulator is occluding an object to be observed. This shows that the identification of a context is dependent on other context as well.

Considering this, we developed an extension for an existing sense-plan-act system by parallelizing perception and action as follows: In the main execution, decisions are made based on perception results. We explicitly declare the so-called *context-variables* that represent this contextual information for decision-making. Then, we develop specific observers to identify these context-variables in parallel to the main execution. In order to be robust against changing situations, the observers depend on additional context decisions that have to be defined and identified. Finally, the context information identified by the observers can be used in the main execution to improve efficiency.

The context-dependence of the observers requires them to implement reactive behavior. Behavior trees provide a suitable structure for this and allow for easy modification and use (Colledanchise and Ogren, 2018).

In summary, we propose an add-on to an existing sense-plan-act architecture to improve the robot’s situation awareness as outlined below:

1. Explicit representation of decision-making context, independent from the main execution.
2. Robust observation of decision-making context by organizing complementary observers in behavior trees. Robustness is achieved by considering additional contextual information about the observability.
3. The above contributions enable the decoupling of decision-making and context observations to increase the efficiency of an existing sense-plan-act system.

**FIGURE 2:**
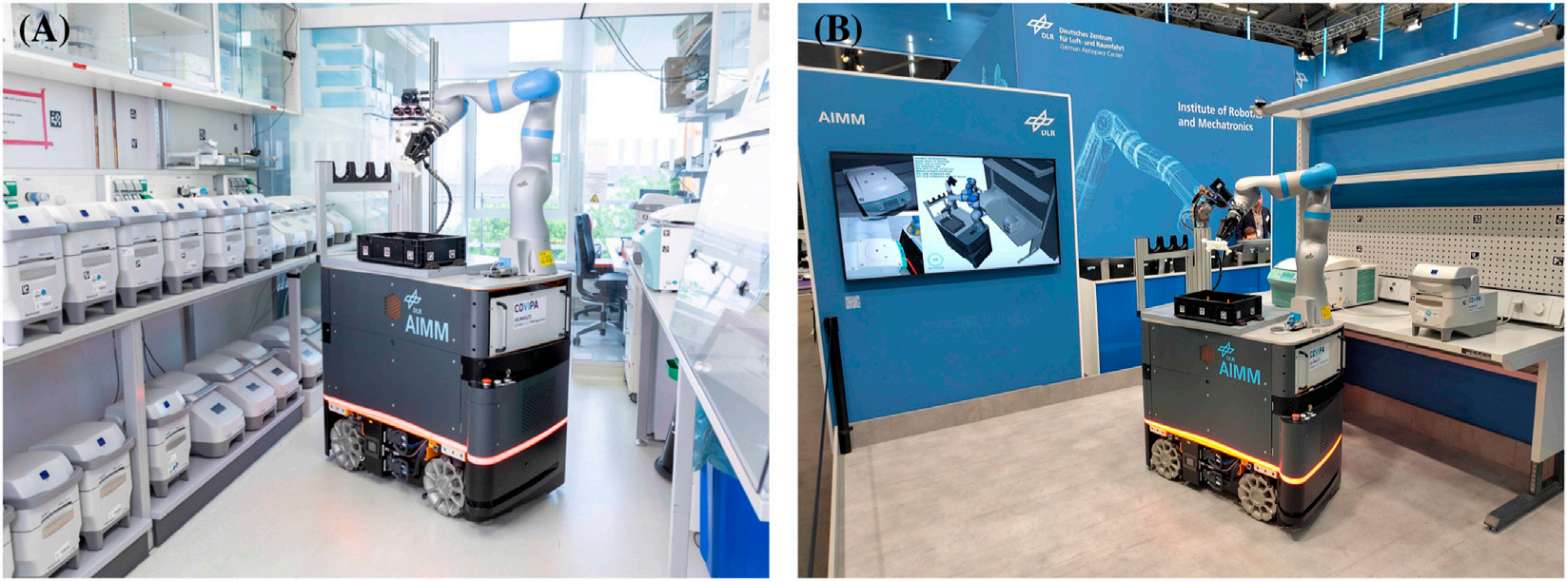
The AIMM system during the task in a real laboratory **(A)** and at the automatica 2025 trade fair **(B)**.

The outlined approach is validated during two major demonstrations in a real medical laboratory and at a large fair of several days (see Figure 2). In the following sections, we first give an overview of the related work in Section 2. Next, the proposed architecture for Context-Driven Robot situation Awareness (CoDRA) is presented in Section 3, followed by experimental validation in Section 4. We conclude with a discussion of our approach in Section 5.

## 2 Related work

Research on improving scene understanding is fundamental for autonomous robots (Graf et al., 2022; Naseer et al., 2019). In this work, we focus on one aspect of scene understanding, namely, situation awareness of the robot. In Smith and Hancock (1995), *situation awareness* is viewed as “generating purposeful behavior in a specific task environment”. In our case, this means making task related decisions.

We define a *situation* as the world state with respect to a fixed point in time (following Müller et al. (2023)). This covers the external state (e.g., object location) as well as the internal state (e.g., process health) of the robot. Some parts of the situation are relevant to the current task, others not. In Sahlab et al. (2022) and Brézillon (1999), context is described as the relevant situational parameters. We further concretize “relevant” by defining *context* as the parts of one or more situations that affect the behavior of the robot. Thereby, it combines relevant situational parameters of different points in time (e.g., with respect to the goal situation).

By previewing and monitoring situations, we aim to detect changes in the scene early, before the corresponding contextual information is needed for decision-making.

The survey by Bavle et al. (2023) highlights the importance of adaptive algorithms for dynamically changing scenes, similar to those in which we deploy our robot. In accordance, Müller et al. (2023) describes situation awareness as monitoring and adaptation of robot behavior to changing situations. After perception, their consecutive step is to reason based on the gathered information. Their concept of improving situation awareness revolves around gathering more contextual information for relevant objects and relations. We take a similar approach when modeling the context explicitly for decision-making and deploying observers in addition to the main execution. However, compared to Müller et al. (2023), which focuses on detecting and handling disruptive events, our main motivation is to increase efficiency and monitor the execution of tasks.

Our method of extracting and identifying the decision-making context to increase situation awareness is also comparable to the approach presented in Dahn et al. (2018). In their work, relevant environmental aspects are identified that can be viewed as our context-variables. According to Dahn et al. (2018), these aspects must be known to achieve situation awareness. While they focus on deriving a concept to measure situation awareness of an agent, our approach introduces a concept to increase efficiency of sense-plan-act systems by situation awareness.

The work of Ruiz-Celada et al. (2024) introduces an approach for a “smart perception system” to better understand the current situation with regard to environmental aspects such as the position of objects. Although we share their motivation to increase situation awareness, the approach is different. While their idea is to collect additional information about the environment with new methods, we focus on parallelizing the perception of existing methods.

Another approach for a flexible perception framework is presented in Mania et al. (2024). They follow concepts proposed with the RoboSherlock framework (Beetz et al., 2015; Bálint-Benczédi, 2020). Behavior trees are used to orchestrate multiple methods of perception (e.g., RGB images, point clouds, and depth images) based on the specific robotic task. In comparison to our work, Mania et al. (2024) uses the logic of the behavior trees to execute the different steps of perception tasks (such as image acquisition, preprocessing, object detection, etc.), while we use it to switch observer behaviors based on the current situation (such as distance to an object or number of available markers).

For robotics in general, we notice that behavior trees are often used as a control structure for the task execution (Mayr et al., 2023; Rovida et al., 2018; Paxton et al., 2017). Using a similar approach but in the application domain of autonomous driving is the work of Forte et al. (2025). Here, behavior trees are used to switch various control and planning strategies based on changing context such as weather conditions or vehicle location. In comparison, we employ behavior trees to make observations parallel to the execution of tasks more adaptive. The observers should deliver correct information independent from the current situation.

The idea of parallelizing perception and action for robotic control architectures has been explored before (Ruiz-Celada et al., 2024; Beetz et al., 2010; Bálint-Benczédi, 2020). An early concept for integrating “active vision” into an autonomous robot architecture was presented by Wasson et al. (1999). We share their central insight that “perception needs to know about action and action needs to know about perception”. Furthermore, they introduce “perceptual memory”, which is conceptually similar to our context manager. In their architecture, perception remains tightly coupled to the skill layer, whereas we scrutinize the decoupling and show what perception can be parallelized, how observations can be performed robustly, and when they are needed. In addition, their work does not address how to orchestrate observers efficiently by activating perception only when a meaningful outcome can be expected.

The work of Ben Ghezala et al. (2014) presents an adaptation of situation awareness in autonomous robotics. They describe an architecture capable of surpassing a blocked situation by modeling situations in an ontology representation. Although their architecture describes continuous perception in parallel to the main execution, the observation of an anomaly is described as a threshold check which does not consider additional contextual information about observability. Similarly to Ben Ghezala et al. (2014), Mayr et al. (2023) also describe a complete robot control framework (SkiROS2) rather than an extension of an existing one. For robust execution, they model the pre-, hold-, and post-conditions of skills. The conditions are evaluated as symbolic predicates over the world model. However, the observation of the world state in parallel to the main execution is not elaborated on. We expect that for the observation of a scene with the SkiROS2 architecture, the use of additional behavior trees as context observers would be beneficial. Since the conditions of skills are already modeled with their proposed architecture, they could directly be translated to declare the context-variables for our approach.

A very recent work that shares our motivation and some of our ideas is presented with the CRESTA architecture in Gasperini et al. (2026). The work describes a cognitivist robot execution framework that supports task-awareness. Similarly to our context-variables, Gasperini et al. (2026) describe so-called “fluents” to represent contextual information about the situation. During action execution, the fluents are observed in parallel and evaluated based on pre-, hold- and post-conditions. The observation of fluents is activated by sending information about the current task to the “perception manager”. Although their architecture allows parallel observers through a plugin system, they do not elaborate on how the observers behave if the observability is impaired. In our approach, we explicitly model these additional contextual conditions for a more robust parallelization of perception. In addition, we evaluate our work in a comprehensive experimental setup that involves a full robotic mission composed of multiple consecutive tasks, extending beyond the single-task experiments presented in Gasperini et al. (2026). Nevertheless, CRESTA provides a modular robotic architecture that could be well compatible with our approach due to its plugin support.

The work of Brunner et al. (2019) addresses general parallelization of robot execution. Therefore, they also parallelize actions like path planning and manipulation in order to increase the efficiency of task execution. To that end, they transform their main execution into a very different structure that can be used for parallel execution. In contrast, the CoDRA architecture can be used in addition to an existing execution, which only needs to be slightly modified. Furthermore, they do not address the problem of limited observability, and parallelization is used only to achieve efficiency. Monitoring of ongoing actions during execution is also not considered. Our approach ensures that observations only take place when adequate conditions (e.g., the object being fully in the field of view) are given. Since the observers are running in parallel to the corresponding actions, context changes can be noticed earlier. After performing an action, the robot will already know whether it was successful or not.

## 3 Methodology for enhanced situation awareness

**FIGURE 3:**
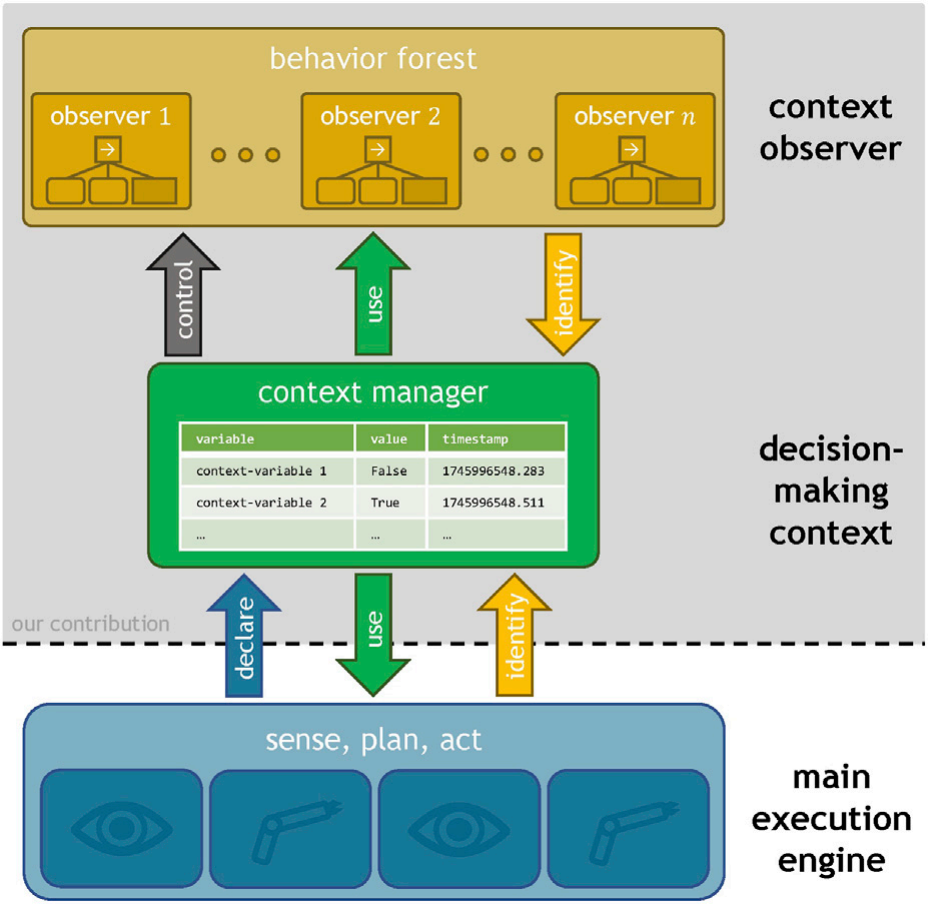
Graphical representation of the general concept, the CoDRA architecture and its implementation. Based on the main execution, context-variables are defined. During runtime, the main execution declares the currently relevant context-variables for the context manager by explicitly creating a named variable with an assigned value and timestamp that represents the decision-making context. The context manager controls multiple observers by starting and stopping them, depending on the current context-variables. The observers identify the value of the context-variables in parallel to the main execution. The identified context-variables are used by the context observers for reliable observations and by the main execution for decision-making. The operations are achieved through interfaces which are implemented for all three major components.

Our goal is to improve the situation awareness of autonomous robots by parallelizing perception. In this section, we describe the proposed approach by introducing the general concept, the architecture, and its implementation as shown in Figure 3.

### 3.1 Concept

The core idea of our approach is to extend a sense-plan-act system with two new concepts: 1) decision-making context and 2) context observers (see right side in Figure 3).

The function of the decision-making context is to explicitly represent relevant aspects of the situation. The entire set of possible context for the robot can be defined as C (Equation 1). This comprises context-variables $c_{i}$ with a unique identifier ${\mathrm{name}}_{i}$ and a value ${\mathrm{val}}_{i}$ holding the information that is relevant to decisions the robot makes (Equation 2).

$$C=\left\{c_{1},\ldots ,c_{m}\right\}$$ (1)

$$c_{i}=\left({\mathrm{name}}_{i},\;{\mathrm{val}}_{i}\right)$$ (2)

Usually, we will model only a subset $C_{M}$ of all possible decision-making context (Equation 3).

$$C_{M}\subseteq C$$ (3)

During runtime, the currently relevant context-variables are *declared* on the basis of the robot’s main execution E , as this is where the decisions on how an action is to be performed are represented. This time-variant set can be defined as Equation 4:

$$C_{E_{t}}\overset{declare}{⟵}E\left(t\right),\;C_{E_{t}}\subseteq C_{M}$$ (4)

For example, the robot’s behavior to manipulate inside a drawer is different if the drawer is open as opposed to if it is closed. The explicit context-variable drawer_is_open can be declared for the decision-making context.

The advantage of this explicit representation is that the value of the context can now be identified independently of the main execution. We use so-called context observers $O_{i}$ for this purpose (Equation 6). Their function is to *identify* the value of one or more context-variables $c_{i}$ in parallel to the main execution (Equation 5).

$$V_{i}=\left\{c_{1},\ldots ,c_{n}\right\},\;V_{i}\subseteq C_{E_{t}},\;|V_{i}|\geq 1$$ (5)

$$V_{i}\overset{identify}{⟵}O_{i}$$ (6)

Identifying context-variables is often dependent on the situation. For example, to check if the drawer is open using a camera image, the drawer must be in the robot’s field of view. This means that the observability of a specific context-variable $c_{i}\in C_{E_{t}}$ can depend on other context-variables. In the main execution, this additional context is usually implicitly established by the previous steps of the execution. In contrast, the purpose of the observers is to identify the value of context-variables reliably in various situations independent from the main execution. To address this, we need to explicitly model these additional context-variables $c_{i}^{\prime }\in C_{O}$ according to all observers (Equation 7):

$$C_{O}=\overset{n}{\underset{i=1}{⋃}}C_{O_{i}}$$ (7)

Based on the values of these additional context-variables $c_{i}^{\prime }$ , the observer has to adapt its behavior and decide if a reliable context value identification is possible. During runtime, a subset of additional context-variables defined for the observers $C_{O_{t}}$ will be organized in the context manager. This can be described as Equation 8:

$$C_{M_{t}}=C_{E_{t}}\cup C_{O_{t}},\;C_{O_{t}}\subseteq C_{O},\;C_{M_{t}}\subseteq C_{M}$$ (8)

To identify the value of these additional context-variables, other observers $O_{j}$ can be used in parallel (Equations 9, 10).

$$V_{j}=\left\{c_{1}^{\prime },\ldots ,c_{k}^{\prime }\right\},\;V_{j}\subseteq C_{O_{t}},\;|V_{j}|\geq 1$$ (9)

$$V_{j}\overset{identify}{⟵}O_{j}$$ (10)

In this way, we build up a system of multiple observers F that complement each other through their shared decision-making context (Equation 11).

$$F=\left\{O_{1},\ldots ,O_{n}\right\}$$ (11)

Some of these additional observers are simple, e.g., by identifying whether an object is in manipulation distance. The work of Graf et al. (2022) affirms the importance of such elementary perception skills for scene understanding, but emphasizes that they are often lacking in state-of-the-art methods.

Finally, the identified values of the context-variables in $C_{M_{t}}$ are *used* by the observers F as conditions for observability and by the main execution E for decision-making (Equation 12):

$$F\left(t\right)\overset{use}{⟵}C_{M_{t}},\;E\left(t\right)\overset{use}{⟵}C_{M_{t}}$$ (12)

### 3.2 Architecture for context-driven situation awareness

Our architecture developed for context-driven situation awareness enables the concepts explained above. In Figure 3 we show the context manager M as the central component. Here, we model relevant aspects of the situation by explicitly declaring context-variables used for decision-making. Both, the main execution and the observers, use the identified context-variables. Therefore, saving the information in a centralized knowledge representation is reasonable.

The context manager also *controls* which observers are running and when they should be stopped. This depends on the progress of the execution, which changes the context. In Algorithm 1, we formalized a procedure to start observers. For stopping observers, a similar algorithm can be applied.

Algorithm 1. Runtime control of observers.

**Input:** Relevant context-variables $C_{E_{t}}$ declared by E , context manager M

1:  **procedure** ActivateObservers( $C_{E_{t}},\;M$ )
2:    **for all** $c_{i}\in C_{E_{t}}$ **do**
3: $C_{M_{t}}\leftarrow C_{M_{t}}\cup \left\{c_{i}\right\}$ ⊳ Add context-variable to context manager
4:       startobserver( $O_{i},\;M$ )
5:    **end for**
6:  **end procedure**
7:  **procedure** startobserver( $O_{i},\;M$ )
8:    **if** observerrunning( $O_{i}$ ) **then**
9:       **return**
5:    **end if**
11: $M.\mathrm{start}\left(O_{i}\right)$

12:   **if** $|C_{O_{i}}|\geq 1$ **then** ⊳ $O_{i}$ has additional context-variables defined
13:       **for all** $c_{i}^{\prime }\in C_{O_{i}}$ **do**
14:         **if** $c_{i}^{\prime }\notin C_{M_{t}}$ **then**
15: $C_{M_{t}}\leftarrow C_{M_{t}}\cup \left\{c_{i}^{\prime }\right\}$

16:           **if** observerexists( $c_{i}^{\prime }$ ) **then**
17:             startobserver( $O_{j},\;M$ )
18:           **end if**
19:         **end if**
20:       **end for**
21:    **end if**
22:  **end procedure**

One challenge is to prevent the context observers from disturbing the main execution. The first aspect here is managing resource consumption in concurrent programming. If parallel processes need the same hardware, they must be prioritized. Therefore, we design context observers in a passive manner. This means that they will not control any robotic hardware but instead passively gather available information, for example, camera images. The advantage is that we do not need to orchestrate the resource management of the respective hardware components. For example, if an observer was trying to move the pan-tilt unit at the same time as the main execution, this could lead to unwanted behaviors. By remaining passive, the observers of the CoDRA architecture should never interfere with the main execution. In addition, the architecture must be able to represent if the value of a certain context-variable is unknown. In this case, or if the observers fail to identify required context-variables for any other reason, the main execution should continue sequentially, as without the CoDRA architecture (compare Figure 1).

**FIGURE 4:**
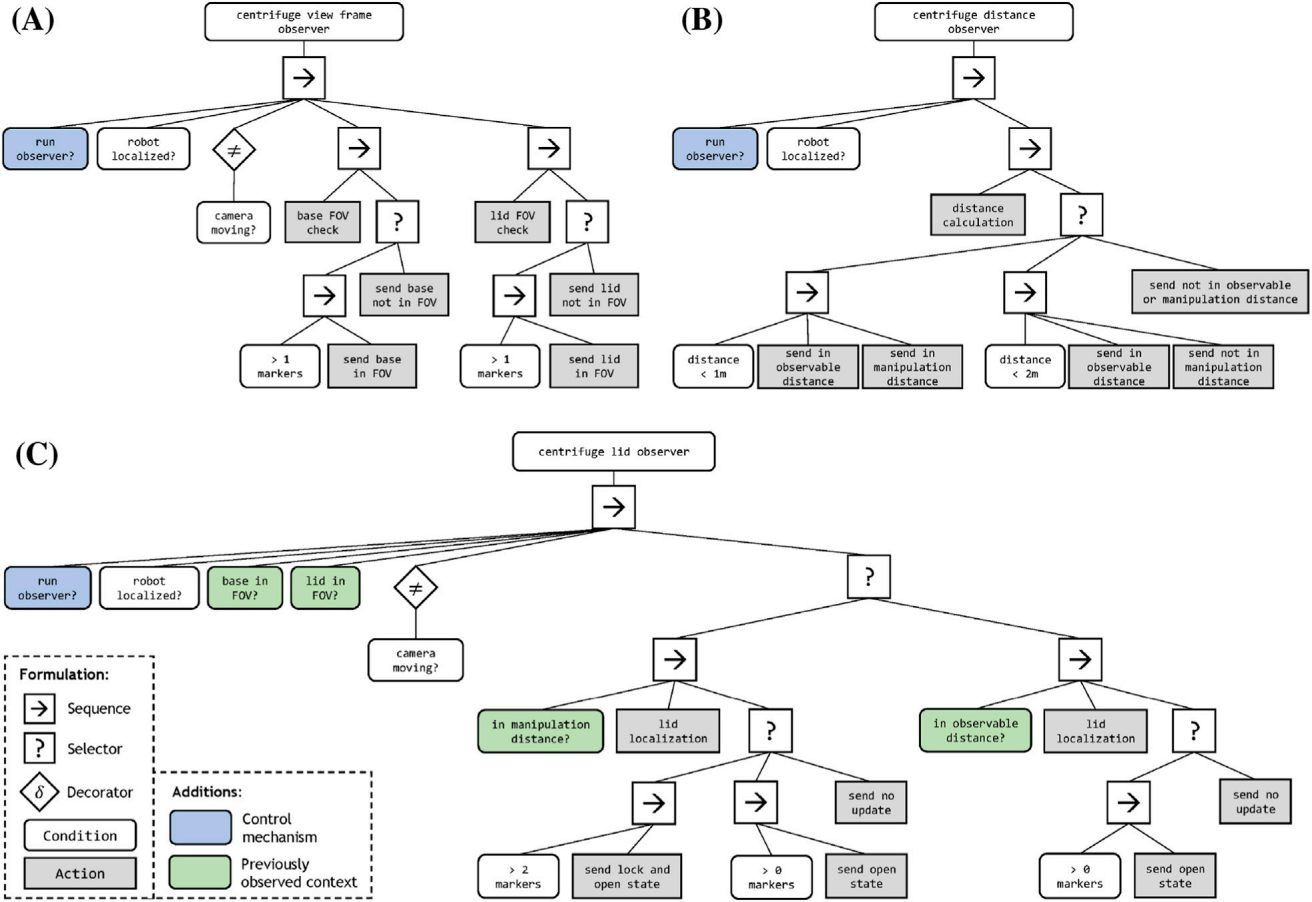
Complementing behavior trees through shared context. A view frame observer checks whether the lid and base of the centrifuge are in the FOV of the camera **(A)**. Another observer determines how close the robot is to the centrifuge **(B)**. Both observed context-variables are relevant for the third observer that determines whether the centrifuge is locked or unlocked and closed or open **(C)**. The conditions marked blue indicate the control mechanism to run the observer. The conditions marked green indicate where the previously observed context is required. The formulation follows Colledanchise and Ogren (2018).

Due to limited observability, resources, and knowledge, observers need to adapt their behavior based on decision-making context that affects the observations. Behavior trees provide a suitable structure for observers as they combine reactivity (through a fixed ticking rate) and explainability (through a clear separation of conditions and actions) in a scalable frame (Colledanchise and Ogren, 2018). While each observer runs as a separate behavior tree, the system of observers forms a “behavior forest” (compare F ) where the execution of one tree depends on the context-variable delivered by other trees. An example of this structure is given in Figure 4, where an observer identifies the state of a centrifuge.

In Algorithm 2, we formalized an approach to build up a system of context observers as behavior forest. The trees that were created with Algorithm 2 can also be combined for efficiency if the identified context-element depends on the same observability conditions (see Figure 4C). Also, additional conditions can be added to the tree to refine the result of the observation behavior and map it properly to the context-element. In the example of Figure 4, this is done by checking the *lid localization* result for the number of available markers. However, this check can also be performed in the perception behavior directly. If no perception algorithm exists to observe the value of a corresponding context-variable, the identification must come from another source. For example, some context-variables might directly be identified by the main execution and do not require an observer (compare arrow in Figure 3).

Algorithm 2. Building a system of context observers as behavior forest.

**Input:** Main execution E

**Output:** Behavior forest F , context manager M

1:  **procedure** buildobserversystem( E )
2: F←∅ , M←∅

3: Π← identifyperceptionalgorithms( E ) ⊳ Where decisions are based on perception
4:    **for all** $\pi_{i}\in \Pi$ **do**
5: $c_{i}\leftarrow$ definecontextvariable( $\pi_{i}$ ) ⊳ Concretize context as context-variables
6:      processcontextvariable( $c_{i},F,M$ )
7:    **end for**
8:    **return** F , M

9:  **end procedure**
10: **procedure** processcontextvariable( $c_{i},F,M$ )
11:    **if** $c_{i}\in C_{M}$ **then**
12:      **return** ⊳ Variable already defined
13:    **end if**
14: $C_{M}\leftarrow C_{M}\cup \left\{c_{i}\right\}$ ⊳ Add context-variable to context manager
15:    **if** hasperceptionalgorithm( $c_{i}$ ) **then**
16: $O_{i}\leftarrow$ createbehaviortree()
17:      addperceptionbehavior( $O_{i},\;\pi_{i}$ ) ⊳ Add perception algorithm $\pi_{i}$ to tree
18: $V_{j}\leftarrow$ defineadditionalcontextvariables( $\pi_{i}$ ) ⊳ Based on observability conditions
19:      **for all** $c_{i}^{\prime }\in V_{j}$ **do**
20:        addpreconditionnode ( $O_{i},\;c_{i}^{\prime }$ ) ⊳ Add additional context-variable to tree
21:        processcontextvariable( $c_{i}^{\prime },F,M$ ) ⊳ Recurse on each additional context-variable
22:      **end for**
23: $F\leftarrow F\cup \left\{O_{i}\right\}$ ⊳ Add behavior tree to observer system
24:    **end if**
25:  **end procedure**

Splitting up the observers into multiple behavior trees has several advantages. We can parallelize the identification of context-variables and hence enable higher efficiency. Another advantage is that trees can easily be controlled by the context manager and activated only when needed.

The described architecture enables us to identify the decision-making context in parallel before using it in the main execution. Therefore, using the CoDRA architecture enhances the situation awareness of our system.

### 3.3 Implementation overview

The context manager holds all context-variables in a central data structure (see the middle part in Figure 3). Context-variables are uniquely distinguished by their name and are saved with their corresponding value. In addition, a flag is added to indicate whether the value of a variable is known or unknown. To keep a history of each context-variable, we save the absolute timestamp when the value is identified. This helps to determine which specific actions were executed by the behavior tree in hindsight. Interfaces to set and retrieve context-variables are exposed to let the context observers and the main execution engine communicate with the context manager. We use ROS2 as middleware and run the context manager in a separate node.

The CoDRA architecture is programmed in Python which allows for an easy integration of the PyTrees library^1^ to control the observers with behavior trees. To separate observers, each runs in an individual ROS-node that manages the tree. We selected a tick-rate of 1 s for the behavior trees which provides a sufficient update rate for the context-variables while allowing to perform all the condition checks via ROS2 within one tick. Although a higher tick-rate would have been possible, we did not deploy the system in a dynamic environment involving frequent external interventions or rapidly changing scene conditions. For our robot, the switching of behaviors is dependent on the underlying skill implementation and usually takes longer than 1 s . Therefore, this tick-rate was sufficient while also not being to resource expensive. As individual actions inside the tree might take longer than the tick rate, we implemented them as asynchronous actions. The corresponding node of the tree will return *running* until the asynchronous action is completed. In combination with behavior trees, the context manager is similar to a blackboard, a common structure for sharing information between nodes (Secchi, 2024).

The CoDRA architecture can be used in addition to the main execution with minor modifications: If a context-variable is needed for decision-making but was already identified by an observer and is therefore known in the context manager, we can skip the upcoming perception task in the main execution and perform a context check instead by querying the context manager (compare Figure 1). If no information is provided by the context manager, the main execution falls back to the default behavior. During task execution, the main execution declares the relevant context-variables for the context manager. In our case, this information is embedded in the skills that we use to control the robot. If the upcoming task is to manipulate a specific object, the main execution publishes this information. Based on the declared context-variables, the context manager can then start and stop the necessary observer for the specific object. In the behavior trees, this is implemented by additional control flags that are checked first at each tick (see Figure 4).

## 4 Experimental validation

In this section, we validate the proposed architecture based on the performance of our autonomous robotic system during two major demonstrations, namely, the CoViPa^2^ campaign and the automatica^3^ 2025 trade fair. The former demonstration is executed inside a hospital medical laboratory, which posed an environment to the robot in a real-world setting and required adaptations of existing skills and modules.

For example, there was a variation between the objects the robot was originally tested with and the ones it needed to manipulate in the medical laboratory (e.g., hinges behaved differently and required adaptation of the manipulation strategies). Also, the robot needed to navigate in a new environment with different lighting conditions, additional reflections, and confined manipulation spaces while managing varying object positions. The demonstration comprises a machine tending task within the scope of a common sample processing workflow. This workflow is representative of routine virological tests, for example, during the COVID-19 pandemic, usually executed by human workers through a series of repetitive manual steps. The robot first has to fetch centrifuge trays with sample containers from a centrifuge and then deliver them to a Polymerase Chain Reaction (PCR)-cycler. Here, the cycler performs PCR to amplify DNA in the samples. After that, the robot retrieves the samples again from the PCR-cycler and delivers them back to the centrifuge to complete one cycle of the workflow. An excerpt of this task can be seen in the supplementary video (see Section Supplementary material).

**FIGURE 5:**
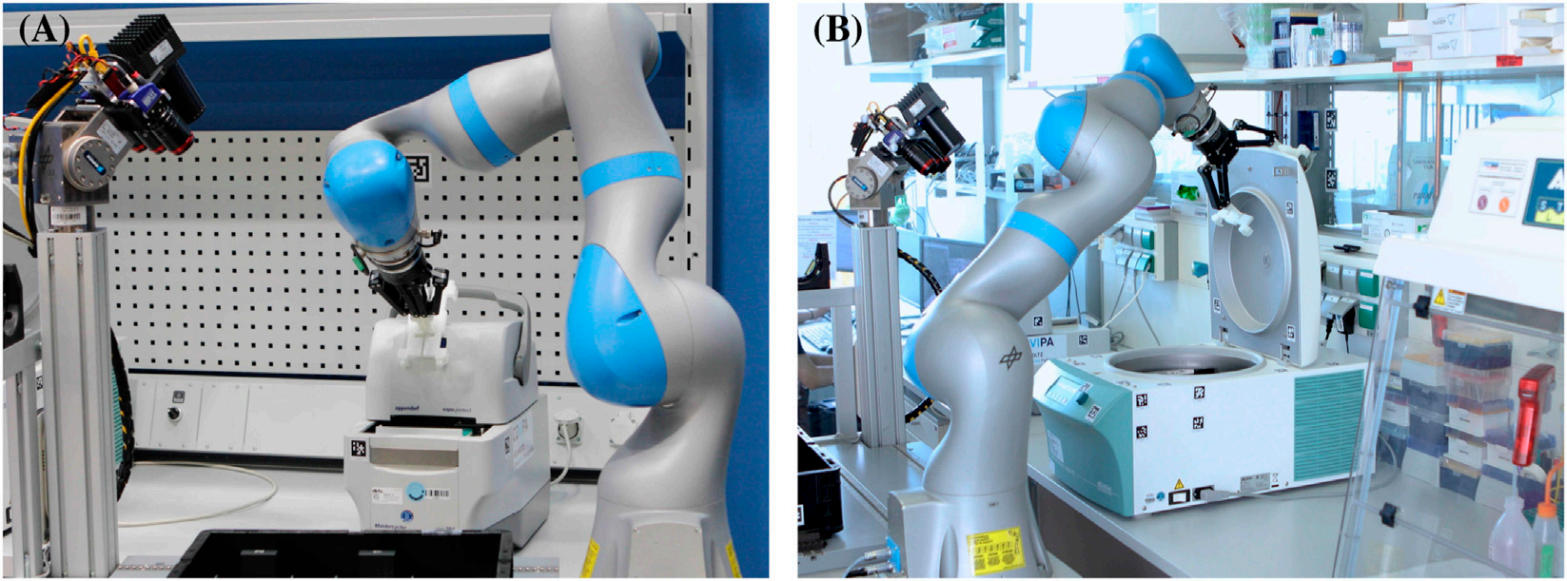
Different machine tending tasks of the robot: Opening of a PCR-cycler after unlocking it **(A)** and closing of a centrifuge **(B)**.

To perform this task, the robot has to manipulate sample containers and other objects, open and close machines, and deliver samples around the laboratory. AprilTags are placed as markers in the environment and on the machines for the robot to orient and locate. As main execution engine, the robot uses the hierarchical state flow system RAFCON (Brunner et al., 2016). It was used to create the robotic mission by orchestrating the underlying skills and manipulation strategies as well as parameterizing the interfaces to the various distributed modules (e.g., the world model or the motion planner). Snapshots of two different machine tending operations that are part of the described autonomous demonstration are shown in Figure 5.

To improve situation awareness and efficiency during this task, we extended the existing cognitive robot architecture of our AIMM (Autonomous Industrial Mobile Manipulator) system. As we view CoDRA as an extension that is agnostic to the underlying architecture, we will not elaborate on the AIMM architecture in this work. However, further information can be found in Dömel (2025), Dömel et al. (2017). For the experiments, we extended the architecture with seven observers to cover different machines and context (see Table 1).

**TABLE 1:** Observers and their respective identified context-variables used for the experiments.

Observer	Identified context-variables	Visualization
Centrifuge distance	*centrifuge_in_observable_distance* *centrifuge_in_manipulation_distance*	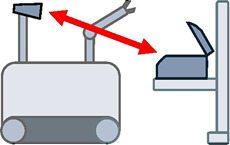
Centrifuge view frame	*centrifuge_base_in_FOV* *centrifuge_lid_in_FOV*	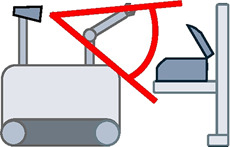
Centrifuge lid	*centrifuge_open* *centrifuge_locked*	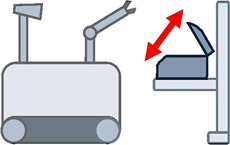
Centrifuge rotor	*centrifuge_rotor_in_pos_1* *centrifuge_rotor_in_pos_2*	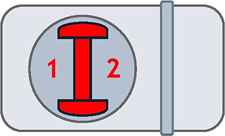
PCR-cycler distance	*PCR-cycler_in_observable_distance* *PCR-cycler_in_manipulation_distance*	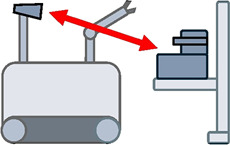
PCR-cycler view frame	*PCR-cycler_base_in_FOV* *PCR-cycler_lid_in_FOV*	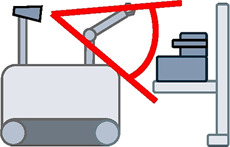
PCR-cycler lid	*PCR-cycler_open* *PCR-cycler_locked*	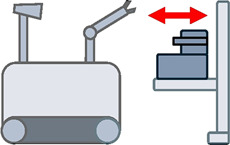

In the following two experiments, we first demonstrate the improved stability of context-driven observers in comparison to a more simple approach where contextual information is not used. Secondly, we show the increased efficiency when using the CoDRA architecture during the autonomous demonstration.

### 4.1 Observer stability through context usage

In the first experiment, we compared our context-driven observers with naive observers that trigger the perception module as often as possible. As an experimental setting, we use the task of the sample processing workflow described above. In addition to the context-driven observers listed in Table 1, we created naive implementations of the centrifuge and PCR-cycler observer. Here, the observer simply runs the lid localization algorithm in a loop, disregarding whether the centrifuge is, for example, in the field of view or even in an observable distance. For this experiment, the main execution is not using the identified context-variables of any observer as we just want to analyze the observer stability.

The demonstration was executed ten times while all observers were running in parallel and identifying their corresponding context-variables. The run continued until the robot successfully completed an entire sample processing loop or encountered an unrecoverable error in the main execution.

**FIGURE 6:**
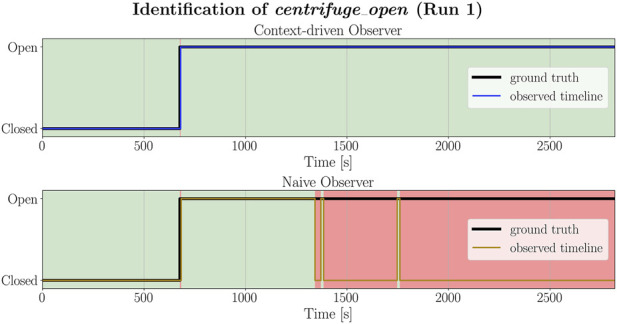
Comparison of a context-driven observer against a naive observer during a demonstration run in a medical laboratory. A red background indicates an incorrect value for the context-variable during that time span. Numerical values are given in Table 2, Run 1.

To evaluate the experiment, we analyze the time spans where the identified context-variables match the ground truth. Over the course of 10 hours of operation in the medical facility, the context-driven observer reached considerably better results, correctly identifying the context-variable *centrifuge_open* over 92.1% of time, compared to the naive observer only over 70.3% of time (see Table 2). For the context-variable *PCR-cycler_open*, we do not find a significant difference in robustness, since both observers deliver the correct result for 99.6% of the time. Taking a closer look at the first run of the experiment, the naive observer incorrectly identified that the centrifuge lid was closed multiple times. This led to a wrong assumption about the current context of the robot over a significant amount of time (compare Figure 6).

**TABLE 2:** Experimental results of 10 hours of autonomous manipulation in a medical laboratory using context-driven (blue) and naive (orange) observers. The lines show the agreement- and disagreement-time in seconds as well as the resulting ratio for a certain context-variable over all runs. Ratios below 0.9 are highlighted red, indicating low reliability of the respective method.

Context- variable		Run 1	Run 2	Run 3	Run 4	Run 5	Run 6	Run 7	Run 8	Run 9	Run 10	Total
context-driven: *centrifuge open*	✓ [s]	2815.11	1393.59	3409.49	1948.05	6362.58	3908.05	1128.22	5468.07	3898.61	2906.36	32929.13
	x [s]	4.26	1.97	4.41	43.17	550.06	2193.74	0.86	2.84	4.88	6.67	2812.80
	ratio	0.998	0.999	0.999	0.920	0.920	0.640	0.999	0.999	0.999	0.998	0.921
naive: *centrifuge open*	✓ [s]	1362.17	1360.12	3378.80	1492.52	3199.46	5422.34	1126.30	1873.83	3585.28	2313.70	25114.52
	x [s]	1457.20	35.44	35.10	498.65	3713.18	679.45	2.79	3597.07	9.22	599.33	10627.43
	ratio	0.483	0.975	0.990	0.750	0.463	0.889	0.998	0.343	0.997	0.794	0.703
context-driven: *PCR-cycler open*	✓ [s]	2807.47	1407.06	3417.81	1981.20	6879.60	6068.58	1126.43	5471.55	3583.64	2919.38	35662.81
	x [s]	15.26	0.00	15.60	14.00	34.21	31.00	0.00	6.23	19.41	7.16	142.97
	ratio	0.995	1.000	0.995	0.993	0.995	0.995	1.000	0.999	0.995	0.998	0.996
naive: *PCR-cycler open*	✓ [s]	2808.33	1407.06	3418.28	1983.57	6877.13	6068.36	1126.43	5467.76	3582.15	2912.21	35651.28
	x [s]	14.41	0.00	15.13	11.77	36.70	31.26	0.00	10.03	20.90	14.33	154.53
	ratio	0.995	1.000	0.996	0.994	0.995	0.995	1.000	0.999	0.994	0.995	0.996

For the context-variable *centrifuge_open*, the context-driven observer is more robust against contextual changes, such as too few visible markers for reliable localization, and considers this when identifying the state of the centrifuge lid. This improved reliability is shown in all runs except *Run 6*, which was caused by a later resolved implementation flaw. Nevertheless, we decided to keep the data from the problematic run for a complete representation of the experiments.

In the case of the PCR-cycler, the detection of the lid and the consecutive estimation of the context-variable *PCR-cycler_open* is easier to observe when no additional contextual information is considered. This is likely because the PCR-cycler is more compact compared to the centrifuge and is modeled with more AprilTags. Usually, more markers will be visible and the whole instrument can be seen in a single image, which is beneficial for the correct identification of *PCR-cycler_open*. However, the use of a context-driven observer is still preferable to a naive observer, as we save resources by only activating vision components when reliable output can be expected. During this experiment, context-driven observers for the PCR-cycler triggered perception components only 1029 times compared to naive observers 5006 times, which is a reduction of 79.4% . This is especially important for an autonomous system as ours, where all operations are run onboard, and the main execution uses the same vision components as the observers.

**FIGURE 7:**
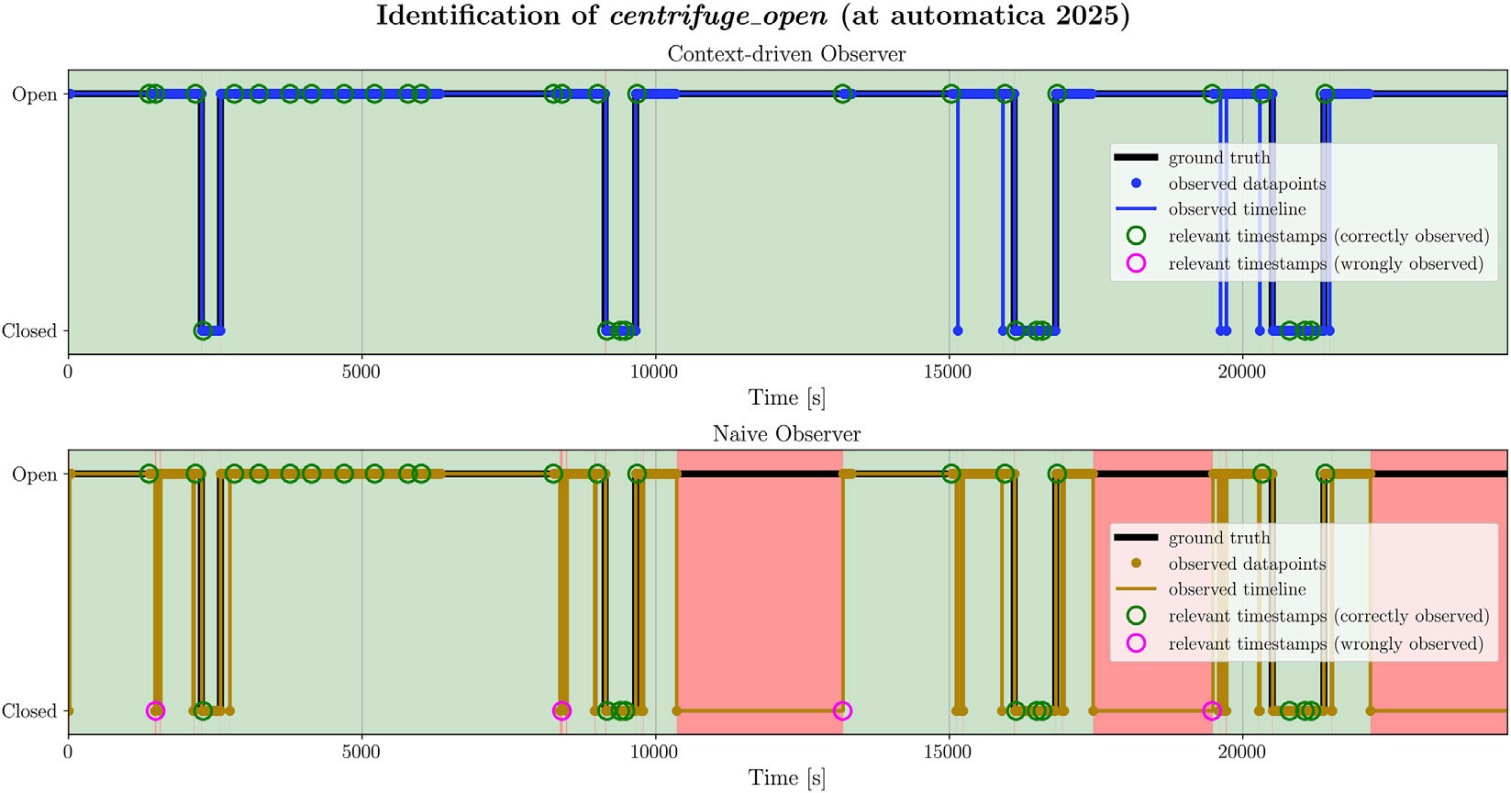
Comparison of a context-driven observer against a naive observer during 1 day of operation at the automatica 2025 trade fair. Rings mark the relevant timestamps when the main execution needs the context-variable for decision-making. A magenta ring indicates that the identified context-variable does not match the ground truth at this point in time. A red background indicates an incorrect value for the context-variable during that time span.

To further demonstrate the improved robustness of context-driven observers, we conducted a follow-up experiment during the automatica 2025 trade fair. Here, the system ran with minor interruptions for a whole workday to demonstrate an autonomous workflow. During the whole operation, context-driven and naive observers were running in parallel. In this experiment, we wanted to investigate if the identified context-variables are correctly identified by the observers at relevant points in time, i.e., when they are needed for the decision-making in the main execution. The results show that the naive observer incorrectly identified the context-variable *centrifuge_open* at four relevant points in time (see Figure 7).

The experiments validate that context-driven observers can reliably identify context-variables in parallel to the main execution. In addition, the approach reduces resource consumption compared to continuous, naive perception triggering.

### 4.2 Reduced runtime through observers

In this experiment, we analyze whether the proposed CoDRA architecture reduces the runtime of the main execution. We focus on the task of opening and closing the PCR-cycler using the robot’s open-object (or close-object) skill. During execution, the robot has to perform checks to detect whether the object is locked/unlocked and closed/open multiple times. The corresponding perception tasks include moving the camera to a suitable configuration, taking an image, updating the object pose, and evaluating it with regard to the task (e.g., deciding whether the object is locked).

Using observers, we can parallelize perception by situation preview and situation monitoring (compare Figure 1). With this approach, the decision-making context can be identified before it is needed for the main execution.

We execute the skill for opening the PCR-cycler five times without and five times with using observers. The experiment is repeated for the task of closing the PCR-cycler.

**FIGURE 8:**
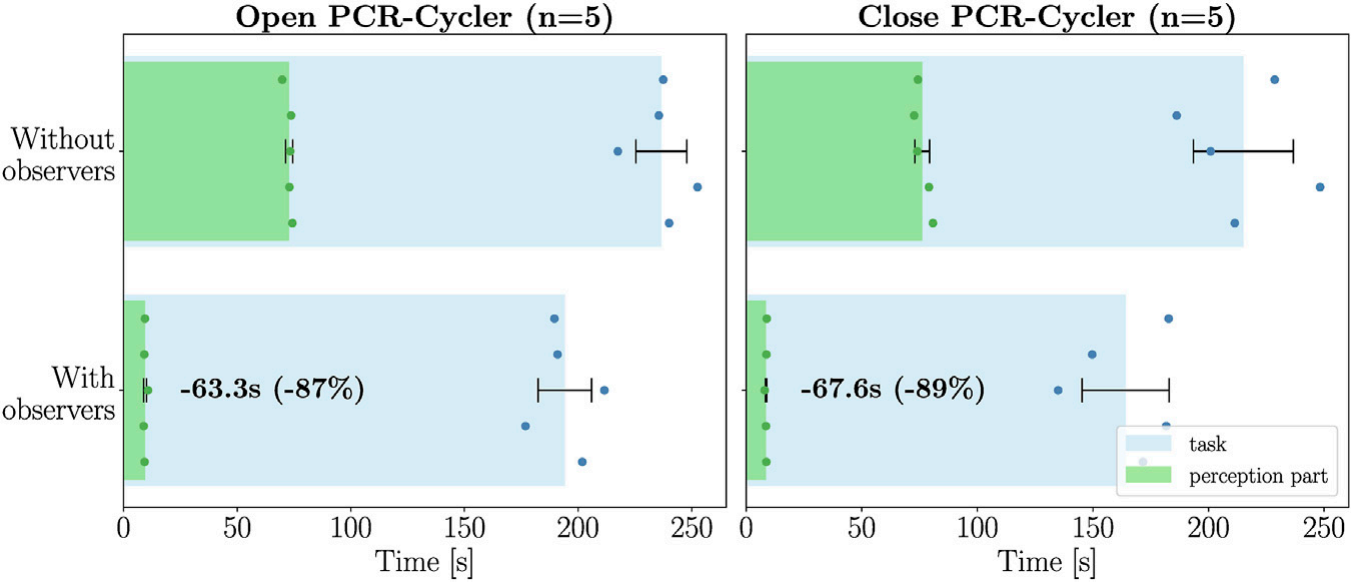
Comparison of execution duration without and with using observers for two different machine tending tasks: Opening (including unlocking) and closing (including locking) of the PCR-cycler. Runtime reduction is shown for the whole task and the perception part.

The results show that the time of perception is reduced by 87% (for opening) and 89% (for closing), respectively, when using observers (see Figure 8). Since every execution includes four perception tasks, the runtime reduction per task is on average 15.8s for opening and 16.9s for closing. This increase in efficiency is also visible when looking at the time spent on the entire task. Here, the runtime is reduced by an average of 18% for opening and 24% for closing. However, a larger standard deviation is visible due to differences in, e.g., planning or motion time.

Using the CoDRA architecture, the main execution can request context-variables from the context manager for decision-making, which takes significantly less time than performing perception tasks. In this experiment, approximately half of the perception time is spent on situation preview and the other half on situation monitoring, as shown in Table 3.

**TABLE 3:** Comparison of the averaged times (n=5) for situation preview and monitoring with and without using observers.

Situation	Observers	Open PCR-cycler	Close PCR-cycler
Preview (2x)	No	37.01s	38.92s
	Yes	4.65s	4.41s
Monitoring (2x)	No	35.79s	37.27s
	Yes	4.86s	4.19s

As an extension to this experiment, we can calculate the total time saved in 7 h of operation during a fair day at automatica 2025. During this time, 153 perception tasks were replaced by context-variable checks for decision-making. On average, 16 s are saved for each perception task, leading to a total time reduction of about 41 min . This confirms that using the CoDRA architecture in addition to our main execution engine increases the efficiency significantly^4^.

## 5 Discussion and outlook

The goal of this work is to enhance the situation awareness of a complex sense–plan–act system, thereby improving its efficiency and robustness. To validate the applicability of the proposed approach under realistic operating conditions, we conducted the experiments within the scope of a full autonomous demonstration in a real medical laboratory rather than creating custom experiments in robotic laboratories. Certain context-variables were central to the decision-making in the robotic tasks. We defined them by analyzing recurring perception patterns in the applied skills. We then developed a specific observer for each context-variable that considers additional context-variables to ensure observability. The additional context-variables were defined with expert knowledge about the perception skill. This limitation can be overcome if the context of robotic skills is already explicitly modeled in the main execution.

For more diverse set of skills, the context-variables would need to be extended too. A current limitation is that the decision-making context needs to be pre-defined offline for the CoDRA architecture. By relying on the context-variables, the adaptation to new robotic tasks requires manual effort. Nevertheless, when the observer setup was extended from the centrifuge to the PCR-cycler, a considerable number of previously declared context-variables could be reused without modification. Fundamental context-variables such as *robot_is_localized* are expected to remain relevant across a wide range of observers and skills. As additional behavior trees are integrated into the behavior forest, an increasing pool of reusable context-variables becomes available. We therefore believe that the proposed concept will scale to larger skill sets, although a systematic evaluation of generalizability was beyond the scope of this work. In the future, we want to test the CoDRA architecture on a different system and evaluate the transferability.

Another important direction concerns observer management under limited computational resources on-board. The topic will become increasingly relevant the more observers are used for context observations. We therefore aim to extend the context manager to prioritize observers dynamically based on task relevance and available resources.

Additionally, the validity duration of certain context-variables should be explicitly modeled based on their dynamics. This would allow the system to focus on the most relevant aspects of the context. Some context-variables require continual monitoring, while for others it is sufficient to observe certain events. The goal should always be to ensure that correct contextual information is used in the main execution while saving on-board computational resources (Lay et al., 2025).

A promising next step would be to integrate CoDRA with an architecture that supports reactive execution in highly dynamic environments. In the current implementation, our evaluation is limited to quasi-static scenarios in which only the robot changes the scene. Extending this toward settings with external disturbances would allow a continued assessment.

In conclusion, our work shows that the explicit decoupling of what, how, and when to perceive has several advantages with regard to stability and performance. In particular, decoupling context observations and decision-making processes in autonomous robots makes their behavior more efficient. Robust parallelization of perception and action can be achieved using context-driven observers.

Instead of creating a new main execution, CoDRA enhances an existing autonomy architecture by increasing the situation awareness. With minor configurations to the main execution and considering only 14 context-variables (see Table 1), we significantly reduced the runtime of the demonstration. We therefore consider our work as a contribution to a range of autonomous systems following the sense-plan-act paradigm.

## Data Availability

The raw data supporting the conclusions of this article will be made available by the authors, without undue reservation.

## Footnotes

1 See *py_trees* GitHub repository (accessed Jan. 15, 2026): https://github.com/splintered-reality/py_trees

2 See CoViPa project (accessed Mar. 31, 2026): https://www.dkfz.de/en/covipa/news/transfer-tandem

3 See automatica homepage (accessed Mar. 31, 2026): https://automatica-munich.com/en/

4 Significance tested on task and perception level using two-sided Welch’s t-test, p<0.01 , n=10 .
